# Clinical performance evaluation of the SiJoy GS1 continuous glucose monitor during oral glucose tolerance testing in healthy adults

**DOI:** 10.3389/fendo.2025.1536292

**Published:** 2025-06-27

**Authors:** Wanyi Zhao, Dongmei Zheng, Xianbao Shi, Feng Xu, Lin Chen, Xingmei Liu, Xiaolong Jin, Qingbo Guan, Chao Xu

**Affiliations:** ^1^ Department of Endocrinology, Shandong Provincial Hospital, Shandong University, Jinan, Shandong, China; ^2^ KeyLaboratory of Endocrine Glucose & Lipids Metabolism and Brain Aging, Ministry of Education, Jinan, Shandong, China; ^3^ Department of Endocrinology, Shandong Provincial Hospital Affiliated to Shandong First Medical University, Jinan, Shandong, China; ^4^ Shandong Clinical Research Center of Diabetes and Metabolic Diseases, Jinan, Shandong, China; ^5^ Shandong Institute of Endocrine and Metabolic Diseases, Jinan, Shandong, China; ^6^ Shenzhen Sibionics Co.Ltd, Shenzhen, China

**Keywords:** continuous glucose monitoring (CGM), plasma glucose, accuracy, glucose control, analytical bias

## Abstract

**Objective:**

This study evaluated the performance of the SiJoy GS1 Continuous Glucose Monitor (CGM) system by analyzing the time lag between plasma glucose (PG) and CGM measurements during an Oral Glucose Tolerance Test (OGTT) in healthy adults. This investigation would elucidate the implications of physiological delay time and optimize technical delays in populations.

**Research design and methods:**

A total of 129 participants wore SiJoy GS1 sensors on their posterior upper arms for at least 48 hours before undergoing an OGTT.

**Results:**

To minimize the Mean Absolute Relative Difference (MARD), two approaches were tested: MARD minimization and minimum deviation match. The demographic characteristics of the participants included a mean age of 37.62 (± 11.21) years, height of 169.84 (± 7.81) cm, and weight of 71.86 (± 18.0) kg. Among them 69.0% were healthy. SiJoy GS1 sensors exhibit an excellent performance of consistency with 96.6% at 20/20% and MARD of 8.01(± 4.9) % at the fasting phase. The consensus error grid results showed 89.22% of all values fell within Zone A, and 100% of values were in Zone A+B collectively. In terms of minimizing Mean Absolute Relative Difference (MARD), at 30 minutes of OGTT, the first method suggested a 15-minute delay while the second proposed a 10-minute average delay time. The latter approach was more suitable due to the less variability in the timing of glucose peaks during the OGTT.

**Conclusions:**

In the study, the SiJoy GS1 sensor exhibited consistent performance. Its accuracy was unaffected by subject characteristics. The application of the minimum deviation match method proved advantageous in reducing the CGM delay time.

## 1 Introduction

Continuous Glucose Monitoring sensors first introduced in 1999, aimed at providing continuous blood glucose concentration measurements over several days continuously, to revolutionize the management of diabetes (1, 2). CGM gained significant clinical adoption in the clinical domain over the last decades. It surpasses traditional self-monitoring methods in diabetic patients by providing detailed information on glucose excursion, such as the prevalence of hypoglycemia and hyperglycemia, and glucose variability under real-world conditions (3). In addition, CGM devices have been found to improve the safety and efficacy of diabetes therapy, decreasing the incidence and duration of hypoglycemia, and minimizing variability in blood glucose (2). With the use of this CGM, an extended glycaemic target range results in patients with either type 1 or type 2 diabetics experiencing less time spent in hyperglycemia and having shorter episodes of hypoglycemia, even nocturnal hypoglycemia (4). These benefits demonstrate the efficacy of CGM devices and position CGM as a potential standard as the standard of care for people with both type 1 and type 2 diabetes (5). Currently, there are various types of CGM devices on the market, each exhibiting varying degrees of accuracy and latency. Considering the high demand for CGM sensors in China and globally, finding a reliable sensor is very crucial for both patients and clinicians.

The mean absolute relative difference (MARD) reflects the performance of the CGM sensors. Where lower values indicate superior accuracy. The clinical practice of MARD values in most CGM sensors ranges from 7.5% to 15.3% (6). CGM values are obtained from the derivation of glucose measurements in the interstitial fluid (ISF), which is calibrated against plasma glucose (PG) (7). Physiological delays between capillary and interstitial compartments between the interstitial and capillary compartments varied among individuals but was estimated to be roughly 6 minutes with a conservative estimation of around 10 minutes. Further, technical time delay could also have been introduced via the sensor (8). Notably, post-OGTT studies report the time lag between PG and ISF after OGTT among 120 pre-diabetes participants was about 10 to 15 min, which was greater than that of the usual physiological and technical delay (9).

OGTT remains the gold standard in evaluating both fasting and post-challenge glucose metabolism. It is used as a gold standard for the diagnosis of prediabetes and diabetes together with Hemoglobin A1c (HbA1c) (10). In one study that included 41 non-diabetic subjects, there was a report of high inter-individual variation in the relative differences between CGM vs PG at 60 and 120 minutes post-OGTT (11). Currently, data are scant on the relation of the values of CGM and PG during periods of rapid glucose rise or fall. Furthermore, only a few studies have assessed the performance of CGM sensors in OGTT. The SiJoy GS1 sensor showed overall excellence in performance consistency: 96.6% at 20/20% and a MARD of 8.01 (± 4.9)% during the fasting phase. It is expected that comparing PG to CGM during an OGTT using the SiJoy GS1 sensor can serve to provide further insights into the role of physiological delay times and thereby contribute to the optimization of technical delays in healthy populations. Besides, the primary aim of this study is to propose and evaluate two methods—MARD minimization and minimum deviation match—for analyzing the time lag between plasma and interstitial glucose levels. Our research could, therefore, potentially advancing clinical utility of CGMs into clinical practices.

## 2 Materials and methods

### 2.1 Participants

The study consisted of 480 healthy adults briefed on the study’s objectives and procedures before giving their written consent to participate. In the final analysis, a total of 129 participants who had complete OGTT time records were included. The inclusion criteria included non-smokers, aged between 20 and 60 years, with no previous history of diabetes and other conditions; not abusing alcohol; no recent or chronic blood donation; BP less than 140/90 mmHg; and no current medication use, including drug abuse, prescription drugs, over-the-counter medication, vitamins, and were not subjected to surgery lately. The study was approved by the Ethics Committee of Biomedical Research Involving Human Beings of Shandong Provincial Hospital and performed in strict accordance with the guidelines of the Helsinki Declaration. Number: SWYX: No. 2021-534.

### 2.2 Study design

In this cross-sectional observational study conducted between December 2021 and December 2023, a CGM sensor, SiJoy GS1 (SiBionics, Shenzhen, China), was attached to the posterior upper arm at least 48 hours before the OGTT trial. The volunteers needed to fast for at least 10 hours overnight and then arrived by 8:00 AM to the Endocrinology Department of The Shandong Provincial Hospital (Jinan, Shandong, China). Then, they Complete health questionnaires upon arrival. Each participant then ingested 75g of glucose dissolved in water within a 10-minute interval. In the OGTT procedures, the blood samples for PG and insulin were collected at 0, 30, 60, 120 and 180 minutes. HbA1c was measured at the fasting stage. All clinical samples were transported to the laboratory using an Automatic Loading System (Timedioc A/S, Tempus 600, SARSTEDT, Denmark). Blood biochemical analysis was conducted using three chemistry analyzers (AU5800, Beckman Coulter K.K, Japan). In the entire study, all adverse events were timely recorded.

Patient and Public Involvement: Patients or the public WERE NOT involved in the design, or conduct, or reporting, or dissemination plans of our research.

### 2.3 SIJOY GS1 continuous glucose monitor

A continuous glucose monitor, SiJoy GS1 (SiBionics, Shenzhen, China), was used to measure interstitial glucose. This device operates by implanting a tiny electrode into the subcutaneous tissue. The electrode was co-immobilized with a glucose oxidation enzyme, which allows the real-time monitoring of interstitial glucose concentrations every 5 minutes using the SiJoy mobile application. The device features hypo- (<3.9 mmol/L) and hyperglycemia (>7.8 mmol/L) alerts so that this can be an assurance for early detection in case their glucose levels suddenly stray out of range unexpectedly.

### 2.4 Data management

The primary goal of this study is to assess the performance of the SiJoy GS1 sensor during conditions when there is a divergence between PG and interstitial glucose during an intensive but standardized glucose excursion. Two methodologies were applied to estimate the total time delay, both physiological and technical: i) MARD Minimization: The sensor measured the ISF glucose, which was subsequently shifted (delayed) by 5, 10, and 15 minutes. The optimal delay was derived based on the minimum MARD at each time shift. ii) Inter-Individual Difference Minimization: We aligned all the post-challenge OGTT measurements, i.e., PG, with the CGM value that provided the minimum absolute difference within a 0–15 minute window after the blood sample was drawn. This 0–15-minute window reflects the expected range of physiological and technical delay between venous plasma glucose and interstitial glucose in healthy individuals (9, 12).

## 3 Result

### 3.1 Demographic characteristics of patients

Among the 129 participants with complete OGTT blood sample records, 69% healthy, 31% excluded due to impaired glucose metabolism. The demographic characteristics of the study population are summarized in **Table 1** below, with a balanced gender ratio, an average age of 37.62 (± 11.21), a mean height of 169.84 (± 7.81) cm, and an average weight of 71.86 (± 18.0) kg. Since the primary objective of this study was to evaluate the performance of the SiJoy GS1 in healthy individuals, participants with impaired fasting glucose, impaired glucose tolerance, or diabetes (31% of the 129 enrolled) were excluded from further CGM performance analysis during the OGTT, leaving 69% classified as healthy for final assessment.

**Table 1:** Characteristics of participants (n=129).

Characteristic	Subgroup	n (%) or Mean ± SD
Age (years)	–	37.62 ± 11.21
Gender	Female	67 (51.90%)
	Male	62 (48.10%)
Age-Gender Group	Female (21–40 years)	42 (32.60%)
	Female (41–60 years)	24 (18.60%)
	Male (21–40 years)	36 (27.90%)
	Male (41–60 years)	27 (20.90%)
Height (cm)	–	169.84 ± 7.81
Weight (kg)	–	71.86 ± 18.0
HbA1c (%)	–	5.52 ± 0.45
Subject Type	Normal	89 (69.00%)
	Impaired fasting glucose	6 (4.60%)
	Impaired glucose tolerance	29 (22.50%)
	Diabetes	5 (3.90%)

Data available for 110/129 participants (85.3%).

### 3.2 Overall accuracy and reliability without processing delay

In the fasting phase, SiJoy GS1 exhibited an excellent performance consistency of 96.6% at 20/20%, with a MARD of 8.01 (± 4.9) % (**Table 2**). The 20/20% refers to ISO 15197:2013 criteria, where 96.6% of CGM readings were within ±20 mg/dL (for PG <100 mg/dL) or ±20% (for PG ≥100 mg/dL) of the reference value. However, MARD increased during OGTT (30–180 minutes), peaking at 14.81%, which may be due to variabilities in peak times of PG among different participants. This also suggests that the CGM values lag behind in time compared with plasma glucose. This would have a great effect on the accuracy and reliability of CGM in dynamic glucose variation.

**Table 2:** Consistency and MARD without processing delay.

Category	CGM Matching Pairs (n)	Consistency (%)			MARD (%)	
		15/15%	20/20%	30/30%	Mean ± SD	95% CI
All	385	64.2	76.6	92.5	14.81 ± 13.35	(13.47, 16.15)
OGTT Time (min)						
0	89	87.6	96.6	100	8.01 ± 4.90	(6.97, 9.04)
30	89	42.7	62.9	79.8	16.75 ± 12.11	(14.18, 19.32)
60	89	62.9	75.3	91	16.07 ± 13.12	(13.29, 18.85)
120	87	63.2	74.7	97.7	16.20 ± 14.91	(13.01, 19.39)
180	78	62.8	71.8	93.6	18.20 ± 16.73	(14.00, 21.99)

The overall MARD values for the clinically and widely used sensors are 13.8% for the Medtronic Guardian 4TM Sensor and 12.4% for the Dexcom G6 sensor (13). The obtained value of MARD for the SiJoy GS1 is 14.81%, and hence comparable to such established sensors. The CGM values measured during the OGTT test are used for determining the MARD value of the SiJoy GS1 sensor. During OGTT, the time of high blood glucose level is radically higher than blood glucose in a normal fasting state. Thus, the measured MARD value is larger compared to that measured by estimating CGM values throughout the full cycle of use. Therefore, this finding points out the accuracy and reliability of the SiJoy GS1 sensor.

**Figure 1:**
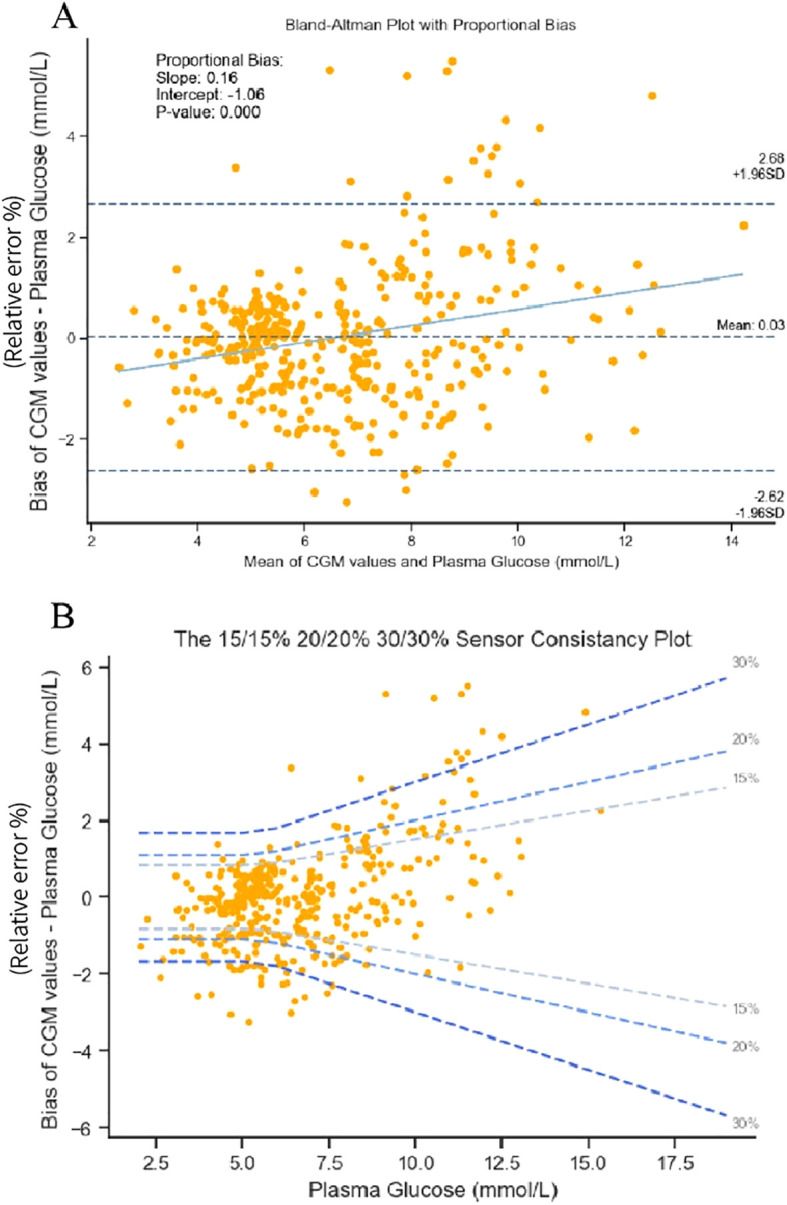
**(A)** Bland–Altman plots comparing blood plasma with continuous glucose monitoring devices for all data points combined (panel A). **(B)** The 15/15%, 20/20%, and 30/30% sensor consistency plot.

To evaluate the consistency between glucose levels derived from interstitial fluid and venous plasma, Bland-Altman plots were constructed for every time point during OGTT, matching the most accurate CGM value (14). Proportional bias was calculated by ordinary least square regression. In **Figure 1A**, 93.2% of points lie within ±1.96 SD limits. **Figure 1A** presents that with the decrease of the blood glucose, the bias becomes smaller, and with the rise of blood glucose, the bias deviates further from the 0-line, and increases. There is a positive correlation between the bias and the mean value of the measurements; hence, with the rise in blood glucose, the error tends to skew upwards, which would reflect underestimation by the sensor. **Figure 1B** shows that 92.5% of the bias was in the 30/30% range and bias increases with an increase in blood glucose level.

### 3.3 Consensus error grid

**Figure 2:**
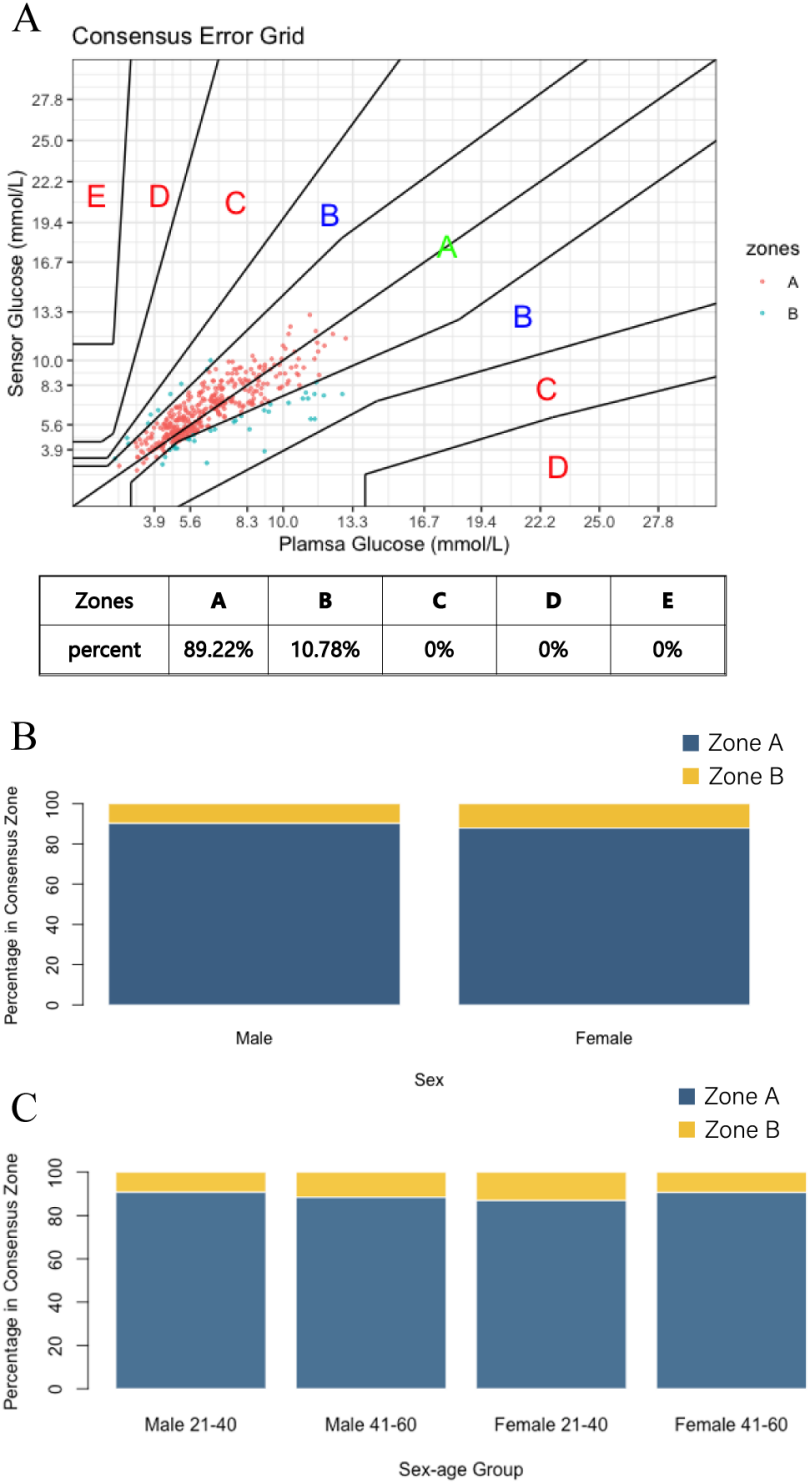
**(A)** Consensus Error Grid of the SiJoy GS1 sensor. **(B)** Percentage on consensus zone of male and female subjects; **(C)** Percentage on consensus zone of different age and gender.

We have also applied a consensus error grid, as recommended by the guidelines for ISO15197:2013 for blood glucose monitoring systems to the data collected from healthy subjects (15). In this study (**Figure 2A**). The results (**Figure 2A**) indicate that 89.22% of all values are located in Zone A, and 10.78% of values are in Zone B, with 100% of values are within Zones A and B. Therefore, the SiJoy GS1 sensor fully meets the requirements per ISO15197:2013 and is safe and accurate enough to be used clinically and in daily use. We further examined the accuracy of the SiJoy GS1 sensor worn by different gender and age subjects in the investigation period (**Figures 2B, C**). Subgroup analyses confirmed consistent accuracy across for different ages or different genders. That means the SiJoy GS1 sensor is suitable for wear by people of any gender and age.

### 3.4 Lag-time between plasma glucose and CGM

**Figure 3:**
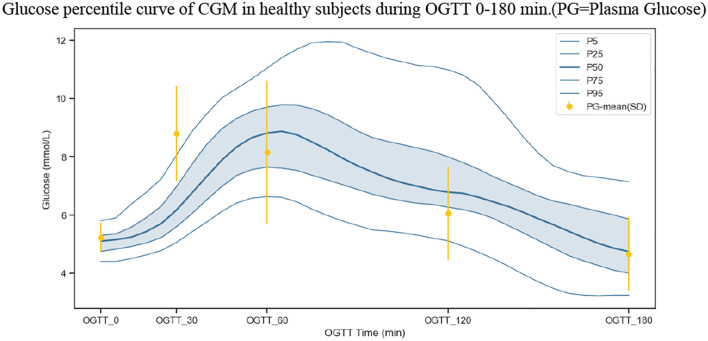
Glucose percentile curve of CGM in healthy subjects during OGTT 0-180 min (P5- 5 percentile smoothed line, P25-P75 inter-quartile range, P50-median, P95-95 percentile smoothed line). Yellow bars represent the mean ± one standard deviation of PG.

As already stated, there is a lag in CGM values relative to PG. In the OGTT experiment, we compared CGM values with PG. **Figure 3** depicts that the peak time of CGM values was somewhat later than that of PG. However, this delay may be negligible in the period of fasting and at 180 min post-OGTT.

#### 3.4.1 MARD minimization

Hence, we employed the method that had the least MARD values to minimize this effect of the delay in CGM values. Once the above method was employed, MARD values were significantly reduced. At 30 and 60 minutes of OGTT, a lag of 15 minutes was observed. Such a lag can be interpreted to be the diffusion time of plasma glucose into the ISF (**Table 3**). And, since the CGM sensor has a high error rate when in a hyperglycemic state, the smaller the measurement values are compared to the PG values. MARD values improved but did still stay high. It showed there is some individual differences.

**Table 3:** MARD minimization and minimum deviation match of CGM values.

OGTT Time (min)			MARD (%)				
			0	30	60	120	180
MARD Minimization	Delay Time (min)	0	8.01	16.75	16.07	16.20	18.20
		5	——	12.90	14.40	14.00	——
		10	——	10.90	12.30	13.30	——
		15	——	9.50	11.90	13.10	——
Minimum Deviation Match	Average MARD (%)	No delay	8.01	16.75	16.07	16.20	18.20
		Delay	——	7.25	8.80	8.25	——
	Delay Time in %	0 min	——	13.50	14.60	20.70	——
		5 min	——	21.30	18.00	23.00	——
		10 min	——	16.90	24.70	23.00	——
		15 min	——	48.30	42.70	33.30	——
Average Delay Time (min)			——	10.00	9.75	8.45	——

#### 3.4.2 Minimum deviation match

Another approach for mitigating CGM value delay was the minimum deviation match. Each subject’s plasma glucose was matched with CGM values that lagged 5, 10, and 15 minutes, respectively, where the CGM value with the lowest deviation was considered as best match (**Table 3**).

In the minimum deviation match method, with the OGTT progression, it was observed that the percentage of 0-minute or 5-minute delay time was increasing and that of 15-minute delay time was decreasing. This suggested that with time, the trend of delay was declining. Indeed, at 30 minutes of OGTT, it has been noticed that the shift from the first method described in this paper was 15 minutes, while for the second method, it was an average of 10 minutes. The minimum deviation method demonstrated superior clinical relevance by the former does not take into consideration that there are individual variations in the timing of the individual peaks of glucose during OGTT. At 60 and 120 minutes of OGTT, the difference between the mean delay times obtained by these two methods was also large, which further points to a significant variation in delay times for individuals for the same reason.

## 4 Discussion

The performance and reliability of the SiJoy GS1 sensor were measured in this study. The SiJoy GS1 CGM system achieves clinically acceptable accuracy between the SiJoy GS1 sensor readings and the plasma glucose. The overall MARD of the product stands at 14.81%, with an impressive MARD of 8.01% at baseline PG levels. The overall MARD values for the clinically and widely used sensors are 13.8% for the Medtronic Guardian 4TM Sensor and 12.4% for the Dexcom G6 sensor. While the Dexcom G7 sensor is now commercially available, the Dexcom G6 sensor was selected for comparison in this study due to its broader current adoption in clinical settings and cost considerations. Subsequent studies may incorporate comparative analyses with the Dexcom G7 sensor for a more updated evaluation. In the consensus error grid analysis, all values fell within Zones A and B reflecting sensor precision (**Figure 2A**). The currently accepted standard of care for monitoring diabetes patients is 4–6 times point of care capillary blood glucose testing, thus resulting in extended periods without glucose value monitoring (16). Continuous Glucose Monitoring systems provide an alternative approach to measuring glucose with the added advantage of greater frequency, measuring every few minutes. This would facilitate the finding of abnormal glucose values more easily continuously and can be used to guide treatment in optimizing diabetes mellitus regimens. Besides, the SiJoy GS1 sensor has eliminated the need for blood glucose calibration. Regarding dietary guidelines for patients, CGM can personally guide patients according to the individual’s unique metabolic needs and glycemic response, which may confer health benefits (17).

A persistent challenge in CGM systems is the delay between interstitial fluid glucose measurements and plasma glucose values. CGM systems measure glucose levels in the ISF, which is indeed different from glucose in the blood. The changes in either compartment would lead to a difference in results before they would settle down and equilibrate after a few minutes. Thus, CGM measurements are time-shifted compared with results from Self-Monitoring Blood Glucose (SMBG) measurements during rapid swings in glycemia. This is due to a delay caused by the diffusion process of glucose across the capillary wall and through the interstitial space to the sensor (18). In our study, we could observe a delay time between venous plasma glucose and interstitial fluid glucose during static but standardized glucose concentration changes. The observed 10–15 minute PG-ISF delay during OGTT aligns with previous findings in prediabetic populations (9, 12). The 0–15 min interval captures most expected delays in CGM systems. Shorter delays (0–5 min) usually occur during gradual glucose changes, while longer delays (10–15 min) are more evident during rapid postprandial spikes. These variations can influence MARD outcomes and underscore the need for individualized delay adjustment. In another similar study recently performed, 15 healthy overweight men underwent an OGTT, and the time to peak glucose was seen to be significantly delayed for interstitial fluid measurements compared to plasma glucose measurements (19). This delay time longer than usual may be explained by the limited speed of glucose that can cross the compartment between ISF and venous plasma. This speed will depend on numerous factors, including the rate of glucose diffusion, the magnitude of concentration differences in various tissues, blood flow, blood vessel permeability to glucose, and acute changes in the release of insulin and glucagon (9).

Additionally, we also found great intraindividual variability in the values of fasting glucose and 2-hour glucose during OGTT. We also followed two methods to address the impact of delays in CGM values. These two analytical methods—MARD minimization and minimum deviation match—have been used in prior CGM validation studies (7–9, 12), and are grounded in established biophysical understanding of glucose diffusion from blood to interstitial fluid. The difference in results using the two methods of delay indicates that there is significant interindividual variability in the diffusion of glucose concentrations from plasma to interstitial fluids during dramatic changes in blood glucose levels. Prediabetes represents an intermediate stage of glucose dysregulation, wherein impaired fasting glucose and impaired glucose tolerance are observed. And about 10% of subjects with prediabetes annually progress to diabetes (20). Post-prandial hyperglycemia (PPHG) can occur in normal individuals and can, therefore, be detected in real-time by CGM sensors (17). Repeated large glucose excursions, such as PPHG, lead to insulin resistance with hyperinsulinemia, which can cause abnormal glucose metabolism (21). Thus, the earlier the detection of PPHG, the earlier the treatment, and healthier would be their lives. However, substantial variations in time to peak glucose suggest diverse response patterns to OGTT in a population of healthy individuals. Whether these different response patterns are related to the emergence and development of pre-diabetes or even diabetes will require further study in the future.

## 5 Conclusions

The SiJoy GS1 sensor demonstrated consistent accuracy (overall MARD 14.81%, fasting MARD 8.01%) independent of subject characteristics, indicating broad clinical applicability. Our comparative analysis of delay-correction methods revealed the minimum deviation match approach’s superiority, as it effectively accounted for inter-individual variations in glucose kinetics during OGTT (average delay reduction: 33% vs fixed methods). Furthermore, the integrated hypo- (<3.9 mmol/L) and hyperglycemia (>7.8 mmol/L) alert system enhances its clinical utility by enabling real-time therapeutic interventions.

## Data Availability

The raw data supporting the conclusions of this article will be made available by the authors, without undue reservation.
